# Infective Endocarditis Presenting as Bilateral Orbital Cellulitis: An Unusual Case

**DOI:** 10.7759/cureus.1350

**Published:** 2017-06-14

**Authors:** Talal Asif, Badar Hasan, Rehman Ukani, Rebecca R Pauly

**Affiliations:** 1 Department of Internal Medicine, University of Missouri Kansas City (UMKC)

**Keywords:** bilateral orbital cellulitis, infective endocarditis, orbital cellulitis, intravenous drug abuse, cavernous sinus thrombosis

## Abstract

Orbital cellulitis is a severe and sight-threatening infection of orbital tissues posterior to the orbital septum. The most common causes of orbital cellulitis are rhinosinusitis, orbital trauma, and surgery. Infective endocarditis (IE) is a systemic infection that begins on cardiac valves and spreads by means of the bloodstream to peripheral organs. Septic emboli can spread to any organ including the eyes and can cause focal or diffuse ophthalmic infection. Ocular complications of IE classically include Roth’s spots, subconjunctival hemorrhages, chorioretinitis, and endophthalmitis. IE as a cause of orbital cellulitis has been described by only one author in the literature. Here, we present a very rare case of bilateral orbital cellulitis caused by IE. Through this case, we aim to create awareness of the potential for serious ocular complications in IE and provide an overview of the management.

## Introduction

In spite of the advances in medical and surgical treatment modalities, infective endocarditis (IE) continues to be associated with high morbidity and mortality [1]. The infection begins on the cardiac valves and spreads via the bloodstream to involve the peripheral organs [2]. Emboli can damage any organ. Ocular involvement in IE is uncommon and may be associated with blindness attributable to septic emboli and endophthalmitis (inflammation of intraocular structures) [3].

Orbital cellulitis is a serious infection of muscles and fat tissue posterior to the orbital septum [4]. The most common cause of orbital cellulitis is rhinosinusitis [4]. Other potential causes are orbital trauma, ophthalmic surgery, infection of the teeth, endophthalmitis, and dacryocystitis [4]. Association of orbital cellulitis and infective endocarditis has been described in the literature once by Bakshi, et al. who noted the association on the retrospective review of magnetic resonance imaging (MRI) of the head of 12 patients [5].

Here, we present a very rare case of a patient admitted with fulminant bilateral orbital cellulitis and cavernous sinus thrombosis eventually diagnosed with IE. There was no evidence of endophthalmitis on ophthalmological examination. Through this case, we advocate consideration of the diagnosis of IE in a patient with bilateral orbital cellulitis so that this critical diagnosis can be recognized in a timely manner. Informed consent statement was obtained for this study.

## Case presentation

A 33-year-old African-American male with past medical history significant for intravenous drug abuse presented to the emergency department (ED) with the chief complaint of bilateral eyelid swelling and loss of vision in the right eye for one day.

The patient had been seen two days prior in the ED when he had presented with right retro-orbital pain. Computed tomographic (CT) scan of the head was obtained that showed no acute intracranial process. He was treated as having cluster headache with prochlorperazine, diphenhydramine, ketorolac, and oxygen and was subsequently discharged. The very next day, the patient began to develop progressively increasing bilateral eyelid swelling, severe retro-orbital headache with subjective chills and fever. He also developed dysphagia and odynophagia, prompting him to return to the ED.

On arrival, the patient appeared sick and in distress. He had a temperature of 102.2°F, heart rate of 110 per minute, respiratory rate of 18 per minute and blood pressure of 109/69 mmHg. On examination, there was bilateral eyelid edema and swelling. He had conjunctival chemosis and bilateral proptosis. Right pupil was non-reactive to light, had complete ophthalmoplegia with visual acuity 20/400. Left pupil was reactive to light, had lateral rectus palsy with visual acuity of 20/30. There was no murmur on the cardiovascular exam and no peripheral stigmata of infective endocarditis. Oropharyngeal, respiratory and abdominal examinations were also unremarkable.

**Figure 1:**
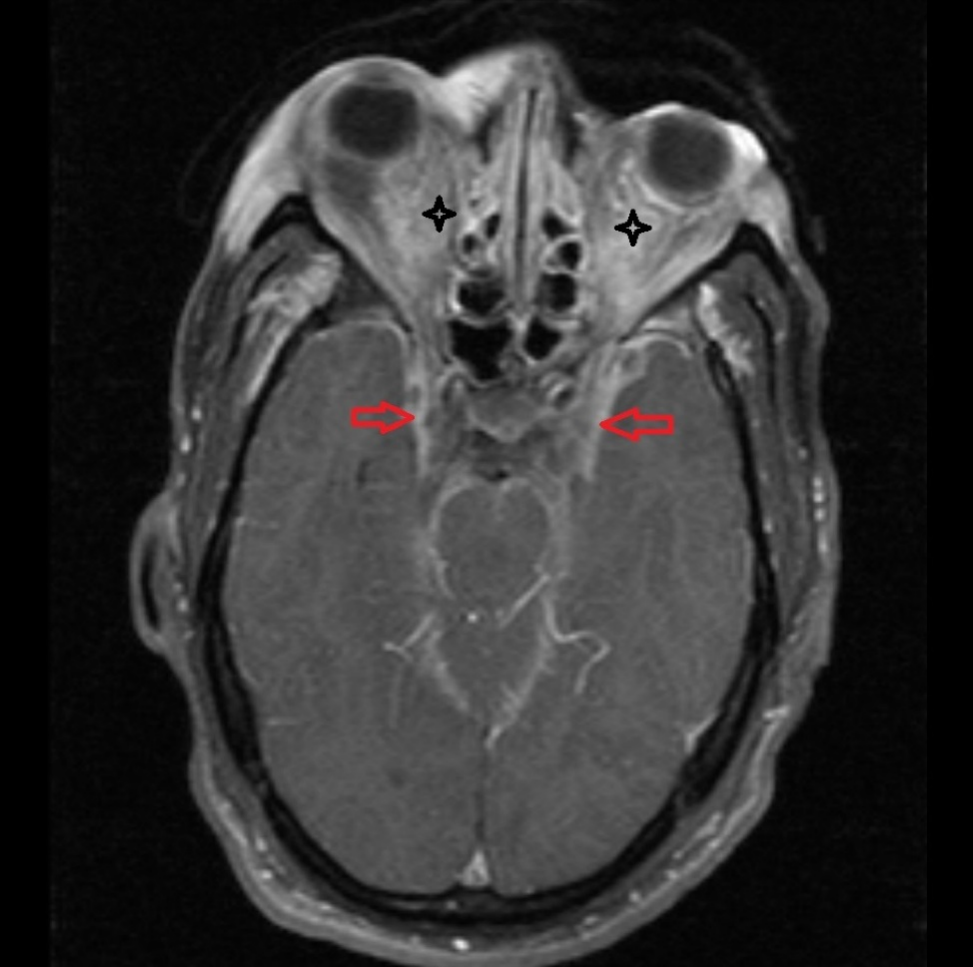
Magnetic resonance imaging (MRI) of the brain showing extensive inflammatory/infectious changes involving the intraconal and extraconal fat of the right and left orbits (black stars) concerning for orbital cellulitis. Meningeal enhancement is also seen at the temporal regions concerning for meningitis (read arrows)

**Figure 2:**
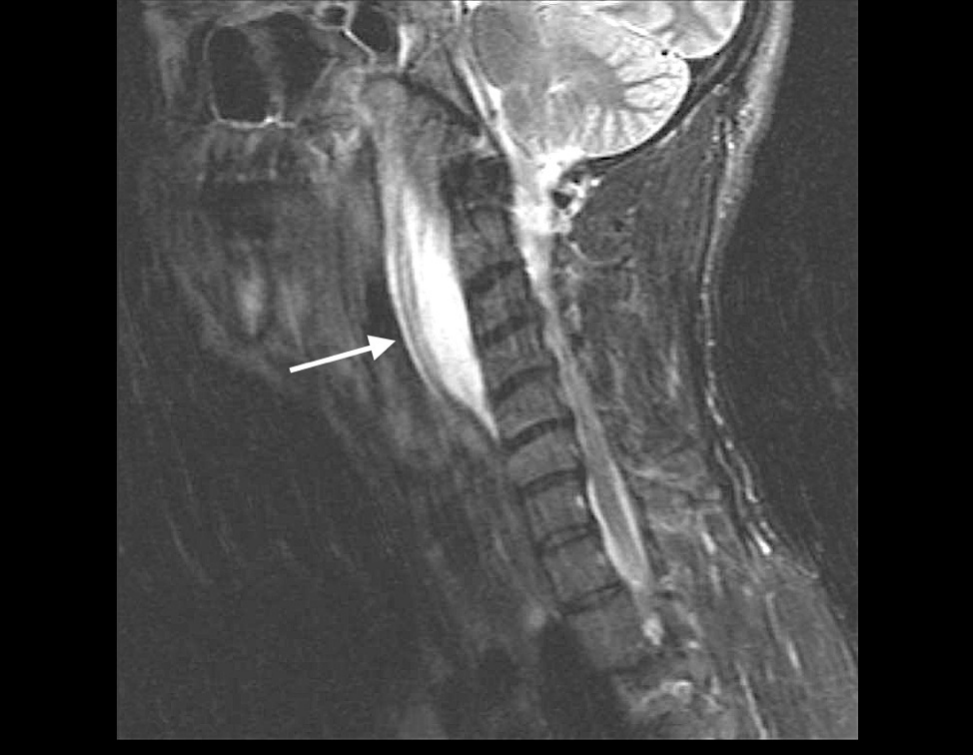
Magnetic resonance imaging (MRI) of soft tissues of the neck showing a retropharyngeal abscess (white arrow)

The initial complete blood count revealed a leukocytosis 19700/cmm with a left shift. The CT scan of the face was then obtained that showed bilateral proptosis with stranding of the surrounding fat concerning for orbital cellulitis. The radiology team also expressed concern about a retropharyngeal fluid collection. Blood cultures were obtained and the patient was immediately started on vancomycin and ampicillin/sulbactam. Infectious disease, otolaryngology (ENT) and ophthalmology consultation were requested. The patient underwent urgent magnetic resonance imaging (MRI) of the brain, orbits and soft tissues of the neck which showed extensive bilateral orbital cellulitis and proptosis (Figure 1), enhancement of the leptomeninges concerning for basilar meningitis and retropharyngeal abscess (Figure 2).

**Figure 3:**
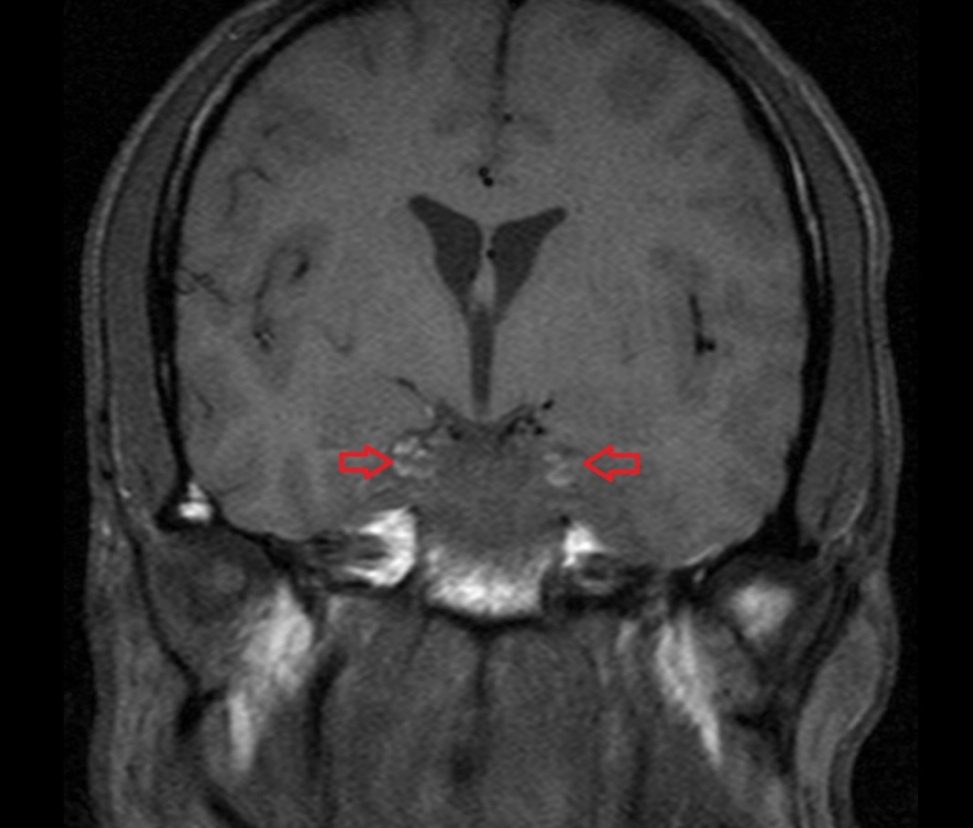
Magnetic resonance venogram (MRV) of the head showing heterogeneous hypoenhancement of the cavernous sinuses concerning for cavernous sinus thrombosis (red arrows)

Magnetic resonance imaging (MRI) brain also showed focal areas of enhancement in the right caudate nucleus and left lateral pons concerning for cerebritis. Magnetic resonance venogram (MRV) showed bilateral cavernous sinus thrombosis (Figure 3).

Magnetic resonance angiogram (MRA) of the head and neck showed normal intracranial and neck vessels.

Otorhinolaryngology (ENT) team took the patient to the operating room emergently owing to the retropharyngeal abscess and concern for airway compromise. He underwent incision and drainage of the retropharyngeal abscess. The patient was then transferred to the intensive care unit (ICU) on mechanical ventilation. He was given then three doses of intravenous (IV) 10 mg dexamethasone every eight hours to decrease orbital inflammation and started on the heparin drip for cavernous sinus thrombosis.

Due to the central nervous system (CNS) findings, lumbar puncture was performed and 20 milliliters (mL) of cloudy cerebrospinal fluid (CSF) was obtained. CSF analysis showed a white cell count of 600 cells/cmm with 94% neutrophils. CSF glucose level was 50 mg/dl and protein level of 135 mg/dl.

**Figure 4:**
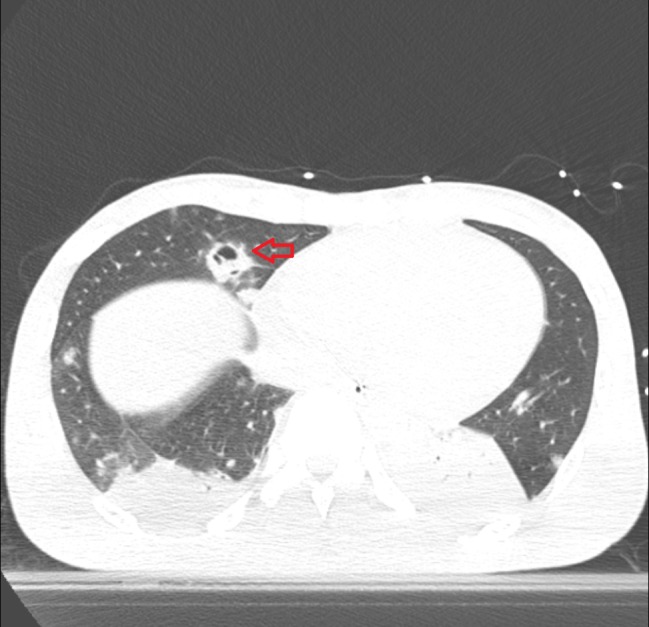
Computed tomography (CT) of the chest without contrast showing multifocal cavitary lesions seen throughout the lungs with largest cavitary lesion (read arrow) seen in the right middle lobe

Patient’s ventilatory support requirements began to increase the next morning. The CT chest scan was obtained that showed multifocal cavitary lesions scattered throughout the lungs concerning for septic emboli (Figure 4).

**Figure 5:**
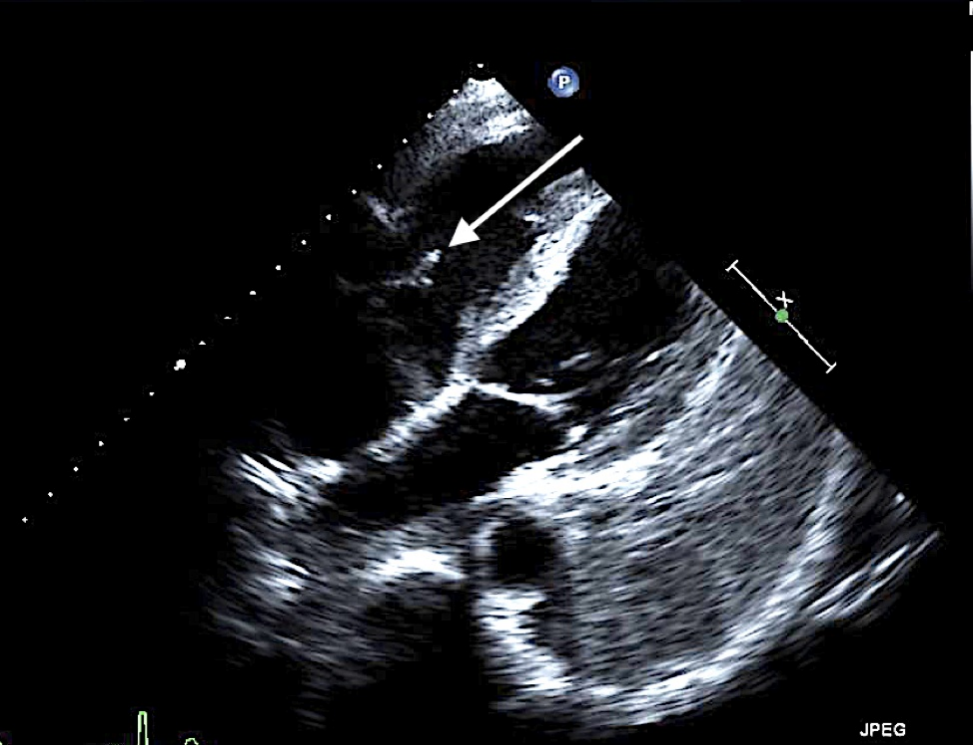
Transthoracic echo cardiogram showing mobile echo density noted on the anterior tricuspid leaflet measuring 0.9 x 0.3 cm (white arrow)

Blood cultures initially obtained in the ED, and the CSF cultures came back positive for methicillin-sensitive Staphylococcus aureus (MSSA). The transthoracic echocardiogram was obtained which showed a mobile echodensity on the anterior tricuspid leaflet (Figure 5).

Antibiotics were switched to IV Nafcillin in the context of identification of the pathogen and for better central nervous system (CNS) penetration.

At this point, it was concluded by the multidisciplinary team that patient’s IV drug abuse had contributed to native tricuspid valve acute infective endocarditis. The vegetation served as the source of infection with hematogenous spread of septic emboli to the orbit, lungs, brain and retropharyngeal space hence accounting for the initial presenting symptoms. With treatment, the patient began to improve and was weaned off the ventilator after one week. Right eye visual acuity improved significantly to 20/50 with complete resolution of ophthalmoplegia. He was subsequently discharged to the long-term acute care facility on oral warfarin and a six-week course of IV Nafcillin.

## Discussion

Infective endocarditis (IE) is a serious infection of the endocardial surface of the heart associated with significant morbidity and mortality [1]. Systemic embolization is a grave complication seen to occur in 22% to 50% of the cases [3]. Clinical manifestations of systemic embolization can precede the diagnosis of IE in 25% to 60% of the cases [6]. Emboli most commonly involve major arterial beds including brain, lungs, spleen, extremities, bowel and coronary arteries [3]. Central nervous system (CNS) and ophthalmologic involvement is a much-feared complication. Embolic events are a marker of poor prognosis in patients with IE [3].

The most common ocular manifestation of IE is Roth’s spots, reported to occur in 2% of patients [7]. These are hemorrhagic lesions on the retina with pale centers. Roth’s spots are a consequence of vascular occlusion by septic emboli followed by a local immune-mediated vasculitis [7]. Other less known ophthalmologic complications of IE include conjunctival hemorrhages, endophthalmitis, and chorioretinitis [7]. In severe cases, blindness may result from endophthalmitis. Bilateral orbital cellulitis as a complication of IE is extremely rare.

Orbital cellulitis is a sight-threatening infection involving tissues posterior to the orbital septum (post septal cellulitis) [4]. Bacterial rhinosinusitis and orbital trauma are the most common causes of orbital cellulitis [4]. Other less common causes include retained foreign body, tumors, mucormycosis, orbital surgery and endophthalmitis [4]. Blood cultures are positive in less than 5% of the cases [8]. The most common culprit micro-organisms isolated are Staphylococcus aureus and streptococci [8]. Orbital cellulitis is associated with a number of serious complications including cavernous sinus thrombosis, meningitis, permanent visual loss and brain abscess [8].

Orbital infection in infective endocarditis was first emphasized by Bakshi, et al. in their retrospective review of magnetic resonance imaging (MRI) of the brain of 12 patients with IE [5]. A total of two patients were noted to have orbital cellulitis with one patient having bilateral orbital cellulitis.

In patients with fulminant bilateral orbital cellulitis and bacteremia, we recommend consideration of the underlying diagnosis of IE. According to American Heart Association (AHA) and Infectious Diseases Society of America (IDSA) guidelines, at least three sets of blood cultures should be obtained from different venipuncture sites with the first and last samples drawn at least one hour apart [3]. Initial transthoracic echocardiogram followed by transesophageal echocardiogram if needed should also be obtained [3]. We also suggest obtaining computed tomographic (CT) scan or MRI of the head to support the diagnosis of orbital cellulitis and identify its complications especially intracranial extension. If cavernous sinus thrombosis is suspected, we recommend computed tomographic (CT) venography or magnetic resonance (MR) venography [9].

We suggest infectious diseases and ophthalmology consultation early in the course of illness. Otolaryngologist consult may also be needed as witnessed in our case. Patients should be started on empiric broad-spectrum intravenous antibiotic therapy targeting Staphylococcus aureus, streptococci, and anaerobes, once cultures have been drawn [9]. The regimen should be revised once the pathogen has been identified and susceptibility results from blood cultures are available. Glucocorticoids may be helpful in reversing inflammatory changes but should be instituted only after antibiotic therapy [10]. Surgical intervention may be needed in patients not responding to medical therapy, ophthalmoplegia or abscess formation [4, 9]. 

## Conclusions

Our case highlights an extremely rare presentation of fulminant orbital cellulitis secondary to infective endocarditis. Rapid diagnosis is critical to prevent mortality. Case reports like this carry immense significance in identifying and providing clinical guidance for physicians about the potential for serious ocular complications of infective endocarditis.
